# Trophectoderm biopsy for preimplantation genetic test and technical tips: A review

**DOI:** 10.1002/rmb2.12318

**Published:** 2020-01-26

**Authors:** Naoki Aoyama, Keiichi Kato

**Affiliations:** ^1^ Kato Ladies Clinic Tokyo Japan

**Keywords:** assisted hatching, blastocyst, non‐assisted hatching, preimplantation genetic testing for aneuploidy, trophectoderm biopsy

## Abstract

**Background:**

Recently, the Japan Society of Obstetrics and Gynecology initiated a clinical study of preimplantation genetic test for aneuploidy. There will be a great need for a standardized embryo biopsy technique in Japan. However, the gold standard trophectoderm (TE) biopsy procedure has not been established, and this review outlines the clinical use of TE biopsy.

**Methods:**

Based on literature, the method and associated techniques for TE biopsy, a dissection method of TE cells from blastocysts, were investigated.

**Main findings:**

Two TE biopsy methods are used, namely assisted hatching (herniating) and non‐assisted hatching (direct suction); however, it is not clear which of these methods is superior. It is critical to understand whether the flicking or pulling method is beneficial.

**Conclusion:**

Non‐assisted hatching biopsy method may cause blastocyst collapse with a higher probability, and it may extend the biopsy time. The biopsy procedure should be performed within 3 minutes, and thus direct TE suction may have greater disadvantages. It is a fact that pulling method of TE dissection with laser pulse is simple; however, excess laser shots may induce a higher frequency of mosaicism. It is important to understand that each technique of TE biopsy has benefits and disadvantages.

## 1 INTRODUCTION

Preimplantation genetic testing for aneuploidy (PGT‐A) was developed as a clinical tool for improving implantation rates and decreasing the risk of miscarriage. However, the efficacy of this technique is still controversial. Higher implantation rates per transfer are expected compared with the control group, but the live birth rate per oocyte retrieval may be lower than in the control group.^1^, ^2^ Thus, misdiagnosis associated with lower diagnostic sensitivity and specificity, and the embryonic damage caused during embryo biopsy are the major disadvantages of PGT‐A. Biopsy technique can be divided into two categories: targeting of the blastomere during the cleavage stage and targeting of the trophectoderm (TE) during the blastocyst stage. Fluorescent in situ hybridization (FISH) protocols require specimens with a wider nucleus diameter on glass slide^3^; this approach is appropriate for blastomere biopsy, which can be useful for obtaining larger nuclei than observed in TE cells. In contrast, more advanced platforms, such as next‐generation sequencing (NGS) and microarray comparative genomic hybridization (aCGH), require optimum DNA from several cells for amplification are appropriate for blastocyst biopsy. TE biopsy for PGT was first reported 15 years ago,^4^ with the development of comprehensive chromosomal screening (CCS) technique, it is becoming a major technique for PGT‐A. The Japan Society of Obstetrics and Gynecology (JSOG) has prohibited PGT‐A screening since 1998; however, in 2016, JSOG initiated a clinical study of PGT‐A for couples with recurrent in vitro fertilization failure and pregnancy loss (Clinical Trials.gov. as UMIN000026104).^5^ In the near future, there will be great need for a standardized embryo biopsy technique in Japan; however, the gold standard TE biopsy procedure has not been established yet. We outline the current status of blastocyst biopsy, and introduce technical tips for practitioners who are studying PGT.

### 1.1 Biopsy method; Hatching or non‐assisted hatching?

**Figure 1:**
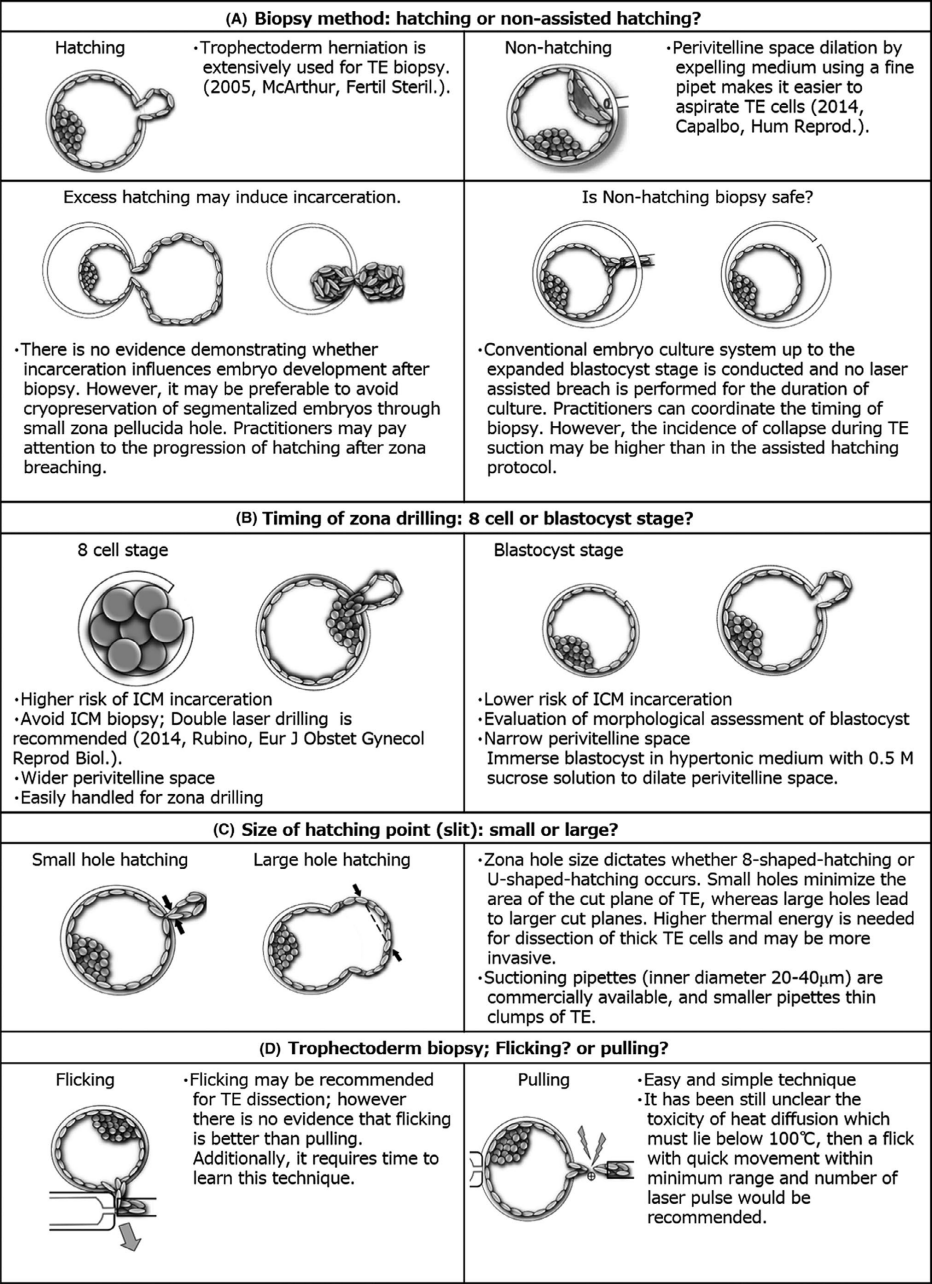
Summary of the critical point of trophectoderm biopsy

The first clinical report of preimplantation genetic diagnosis (PGD) using cleavage‐stage embryo biopsy was published by Handyside in 1990.^6^ At the same time, researchers began studying trophectoderm biopsy and demonstrated the feasibility of this method for PGD.^7^, ^8^ Initially, cleavage‐stage embryo biopsy predominated,^9^, ^10^, ^11^, ^12^, ^13^ particularly that using FISH protocols.^14^, ^15^, ^16^, ^17^, ^18^, ^19^, ^20^, ^21^, ^22^, ^23^ The first live birth case following blastocyst biopsy for PGD was reported in 2002.^24^ McArthur et al^25^ described the details of trophectoderm biopsy procedure and reproductive outcomes of PGD using this technique with FISH or real‐time quantitative polymerase chain reaction (qPCR) in 2005. Notably, in their approach, zona breaching was performed on day 3 or 4, and 2‐9 cells from the herniated trophectoderm were teased free from the remaining trophectoderm with laser pulse.^25^, ^26^ This protocol was frequently applied for clinical trials; however, researchers also proposed another approach, that is, non‐assisted hatching method. Capalbo et al^27^ reported a direct aspiration technique for biopsy of the trophectoderm without assisted hatching in 2014, demonstrating the benefits of this protocol in daily laboratory work. Because the timing of biopsy for hatching blastocyst is critical, excess herniation of trophectoderm may induce incarceration (Figure 1A). There is no evidence for the association between embryo incarceration and embryo development. However, practitioner should still avoid causing excess trophectoderm herniation. Continuous observation using time‐lapse monitoring technology and direct TE aspiration with non‐assisted hatching are helpful for detecting and preventing such early incarceration. Non‐assisted hatching has disadvantages. For example, if blastocyst collapse occurs during TE cell aspiration, the biopsy procedure may be difficult and be associated with a higher risk of losing the inner cell mass (ICM) area. Furthermore, blastocyst collapse may extend the biopsy time, despite recommendations that the biopsy procedure should be performed within 3 minutes.^28^ No published clinical data have shown which method is better for biopsy.

### 1.2 Optimal timing of zona drilling

**Figure 2:**
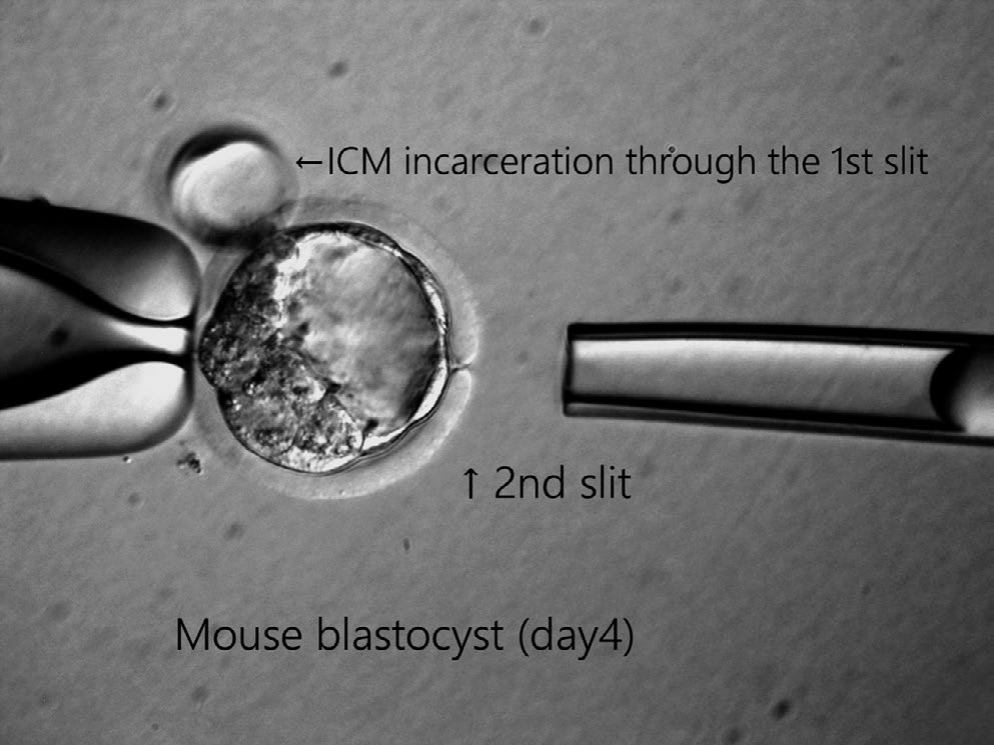
Double zona drilling

When establishing standard approaches for trophectoderm biopsy,^25^, ^26^ it is essential to consider the timing of zona breaching during the cleavage or blastocyst stage (Figure 1B). Zona breaching is associated with a risk of ICM incarceration, and practitioners must be careful to avoid physical damage to the ICM during the biopsy procedure. Zona breaching during the cleavage stage could increase ICM incarceration; however, few studies have reported this phenomenon. Rubino et al^29^ reported a double zona drilling method as a coping technique for ICM incarceration with an incidence of 3.0% (6/199) (Figure 2). Additionally, Gu et al^30^ reported an incidence of 5.5% (590/10 730) in human blastocysts. In contrast, animal data showed incidence rates of 44.2% (42/95)^31^ and 38.6% (49/127; N. Aoyama, Kato Ladies Clinic, unpublished data) for ICM incarceration with zona breaching during the cleavage stage, indicating large differences between human and mice. Importantly, animal experiments are performed using time‐lapse monitoring, whereas clinical data do not always include such detailed information. Furthermore, 52.5% (5633/10 730) of cases were fully hatched blastocysts, which could be included many cases of ICM incarceration.^30^ Onodera et al^31^ suggested that the location of 8‐shaped hatching influences ICM formation in mouse blastocysts, however, this concept is still controversial. Indeed, clinical data have shown that ICM incarceration does not increase monozygotic twinning delivery.^30^ If a part of ICM is aspirated together with TE cells during biopsy, although it depends on the number of TE cells aspirated, several cells reduction from the ICM may not have a huge effect on the embryo development after biopsy. This is because it has been hypothesized that ICM splitting may generate monozygotic twining during the repeat of collapse and expansion in the process of blastulation in a clinical study with time lapse,^32^ or in an 8‐shaped hatching animal experiment.^33^ However, not only the number of cell compensation, but also the chorionicity and amnionicity are also very important for normal fetal development after embryo splitting.^34^ Moreover, cell fate has already been determined at the blastocyst stage^35^ and ICM is formed by two cell lines, which are epiblasto and primitive endoderm in early blastocyst stage.^36^ Therefore, full attention should be paid to TE biopsy, and the double laser zona drilling may be effective to avoid impact on ICM.

Additionally, zona breaching during the cleavage stage may induce herniation in early‐stage blastocyst, which are thought to have fewer cells. Although the total cell number in blastocysts have not been fully elucidated, in 1989, Hardy et al^37^ showed that mean numbers of TE cells were 37.9, 40.3, and 80.6 at 5, 6, and 7 days after insemination, respectively, In contrast, another study by Fong et al^38^ reported that the cell numbers ranged from 160.9 to 236.7 in morphologically high‐quality blastocysts and from 43.7 to 84.0 in poor‐grade blastocyst on day 6 morning. Additionally, a recent report showed that the cell number were 225.2 and 121.1 in hatching and non‐hatching blastocyst.^39^ Thus, there may be greater differences among grades of blastocysts and time after insemination, and these differences may be products of the laboratory environment (incubator, culture medium), resulting in variations in embryonic developmental speeds. In either case, balancing the number of cells biopsied per total cell number may be important for applying TE biopsies, and obtaining full‐size blastocyst may minimize invasion from biopsy. Moreover, continuation culture to full blastocysts may have additional benefits in evaluating morphological grading. Overall, zona breaching on days 3 to 4 after insemination may results in a higher risk of ICM incarceration with fewer benefits compared with zona breaching on day 5 to 6, thereby producing full blastocysts.

### 1.3 The ideal procedure for trophectoderm biopsy

#### 1.3.1 An appropriate number of biopsied TE cells is required to maintain implantation potential

The number of biopsied cells is one of the most critical factors affecting TE biopsy,^40^ the general consensus among researchers is that this number may be 5 cells.^26^, ^41^ However, it is difficult to apply this in the clinical setting, and attempts have been shown to result in poor‐grade TE and fragmentation. Fewer cell biopsies may be less invasive but result in a higher risk of amplification failure, whereas increased cell biopsies may lead to a lower risk of amplification failure but increase blastocyst disruption. Several clinical reports have described the incidence of DNA amplification and inconclusive results using a CCS device, at rates of 12.5% and 4.5‐8.2% by polar body biopsy,^42^, ^43^ 2.9%‐12.3% and 0%‐18.6% by single blastomere isolation,^44^, ^45^, ^46^, ^47^, ^48^ and 0%‐4.0% and 0%‐4.6% by TE,^28^, ^49^, ^50^, ^51^, ^52^, ^53^, ^54^, ^55^ respectively (Table 1). Blastocyst biopsy results in lower amplification failure rates because of the superior cell numbers obtained from biopsy comparing with that of polar body or blastomere biopsy. Cimadomo et al showed that 8.0 trophectoderm cells were required for successful DNA amplification and conclusive diagnostic results; thus, the most suitable cell number for biopsy may be 5‐10 cells rather than 1‐5 cells. Overall, the use of these invasive techniques for TE biopsy may reduce the rate of live births by 5% live births.^56^

**Table 1:** The incidence of amplification and diagnostic failure using biopsied cell and reproductive outcome with PGT‐A

Outcomes (WGA and diagnostic)					Reproductive outcomes					
					PGT‐A group			Control group		
No. of cells	Amprification method	Amplification failure	CCS device	No available result	Age	Result	Age	Result	*P*‐value	Reference
Biopsy sample: PB1/PB2										
1‐2	SurePlex	N/A	aCGH (24sure V3)	4.5% (24/530)	39.5	LB/transfer 26.4% (29/110)	38.4	LB/transfer 14.9% (60/403)	.015	2015, Feichtinger, PLoS One.
1‐2	SurePlex	12.5% (128/1023)	aCGH (24Sure)	8.2% (84/1023)	38.6	Pregnancy/1st transfer 38% (57/149) Cumulative LB rate/Pt. 24％ (50/205)	38.6	Pregnancy/1st transfer 32% (54/171) Cumulative LB rate/Pt. 24％ (45/191)	.71	2018, Verpoest, Hum Reprod.
Biopsy sample: Blastomere										
1	DOP‐PCR	12.3% (9/73)	metaphase‐CGH	0.0% (0/64)	N/A, a validation report using surplus embryos					2000, Wells, Mol Hum Reprod.
1	①GenomePlex ②SurePlex	11.2% (18/161) 2.9% (19/654)	aCGH (BlueGnome) aCGH	3.5% (5/143) 2.5% (16/634)	37.5	Pregnancy rate 57.9%/BT (44/76) 42.3％/OR (44/104)	N/A	N/A		2011, Gutierrez‐Mateo, Fertil Steril.
1	MDA	5.5% (5/91)	aCGH (8K)	18.6% (16/86)	N/A, a validation report using surplus embryos					2013, Mertzanidou, Hum Reprod.
1	PicoPLEX WGA	N/A	Ion PGM Sequencing	8.3% (21/252)	34.0	Implantaion rate 61.5% (40/65)	34.4	Implantaion rate 34.8% (31/89)	.001	2015, Lukaszuk, Fertil Steril.
1	Sureplex	N/A	aCGH (24sure arrays V2)	2.8% (15/538)	38‐41	Implantaion rate 52.8% (47/89)	38‐41	Implantaion rate 27.6% (48/174)	<.0001	2017, Rubio, Fertil Steril.
Biopsy sample: Trophectoderm										
5	qPCR	0% (0/71)	N/A	1.4% (1/71)	N/A, a validation report using cell lines					2012, Treff, Fertil Steril.
5	qPCR	N/A	N/A	1.4% (7/483)	32.2	Implantaion rate 79.8% (107/134)	32.4	Implantaion rate 63.2% (103/163)	.02	2013, Scott, Fertil Steril.
6‐10	SurePlex SurePlex	1.5% (3/195)	①aCGH (24sure V3) ②NGS (VeriSeq)	6.3%* (12/191)	39.9	Implantaion rate 64.0% (32/50)	N/A	N/A		2014, Fiorentino, Hum Reprod.
3‐5	SurePlex SurePlex qPCR	1.4% (6/418) 2.1% (9/427) 1.2% (30/2586)	①aCGH (24sure) ②NGS (HiSeq 2000) N/A	0.0% (0/418) 0.0% (0/427) 4.6% (119/2586)	35.5 35.2 39.4	Implantaion rate 66.2% (88/133) Implantaion rate 70.2% (92/131) Implantation rate 53.4% (264/494)	*P* = .56	N/A N/A N/A		2015, Yang Z, BMC Med Genomics. 2015, Capalbo, Hum Reprod.
5‐10	PicoPLEX	N/A	aCGH (SurePrint G3)	1.3% (3/224)	28‐44	Implantaion rate 42.6% (26/61)		N/A		2016, Liu M, Reprod Biol Endocrinol.
2.1‐7.5	qPCR	2.0% (176/8990)	N/A	0.5% (52/8990)	38.5	LB/transfer 38.8% (19/49)		N/A		2018, Cimadomo, Hum Reprod.
TE:5‐10 ICM:1‐10	SurePlex	4.0% (4/99)	②NGS (VeriSeq)	0.0% (0/95)	N/A, a validation report using surplus embryos					2018, Chuang TH, Mol Hum Reprod.

Abbreviations: CCS, comprehensive chromosomal screening; LB, live birth; MDA, multiple displacement amplification; N/A, not applicable; Pt. patient; WGA, whole genome amplification.

* It is as a non‐concordant rate in the 2 methods.

#### 1.3.2 When should we perform TE biopsy?

No conclusion has yet been reached regarding the best timing of TE biopsy, and we interpret that a point of contention may be the number of biopsied cell per the total cell number. The day 3 zona opening method may prompt hatching in early‐stage blastocysts, which have 40‐60 cells. On the other hand, zona opening in developing or developed blastocyst stage, which have 60‐100 cell can avoid hatching in blastocysts with a fewer cell number. Goossens et al^57^ reported that 16 the removal of one or two cells of blastomere in cleavage stage had a significant influence on embryo development on Day 5, that is; removing a smaller number of cells by embryo biopsy is less invasive for embryo development. Namely, removing 5 to 10 TE cells by biopsy may be less invasive for expanded blastocyst, which have a larger number of total cells than early‐stage blastocyst, which have fewer cells. Actually, we performed TE biopsy when the blastocysts reached full size (>160 µm) with the hatching method in the pilot study of PGT‐A,^5^ because there is no way to assess the total cell number.

On the other hand, from the results of our clinical study, blastocysts, which have earlier developmental speed, showed better reproductive outcomes such as clinical pregnancy and live birth rate^58^; meanwhile, smaller size blastocysts (<160 µm) until day 7 had little chance to achieve clinical pregnancy. We hypothesize that biologically high‐potential embryos reaching full‐size blastocyst until 130 hours after insemination have a higher chance to be euploidy, similar to the report that day 7 blastocysts showed a lower euploidy rate.^59^, ^60^ Finally, the timing of biopsy may be better in the developed or developing blastocyst, which have larger cell numbers. Additionally, if an embryo has an earlier developmental speed, such as developed blastocyst in day 5, favorable result may be obtained.

#### 1.3.3 Size of hatching point and time length to perform the biopsy

In general, single TE biopsy should be performed within 3 minutes.^28^ This can be achieved using a simple biopsy. However, thick TE samples may be difficult to dissect from blastocysts. Thinning the TE area of the cut plane makes biopsy less difficult; thus, we suggest that smaller zona holes may produce less constriction of the hatching site for micro‐cutting (Figure 1C). However, this process may be more complicated. Indeed, if a practitioner ignores the optimal timing of biopsy, a large 8‐shaped hatching site may be generated, resulting in incarceration (Figure 1A). To prevent this, a time‐lapse system may be useful. Furthermore, the size of the suction pipette for TE may be altered to minimize the area of the cut plane; several suction pipettes with inner diameters of 20‐40 μm have been developed and are available from commercial sources.

#### 1.3.4 Trophectoderm biopsy technique using flicking or pulling

The first solid‐state laser to be used for ART, which was applied to trap spermatozoa, was neodymium:yttrium–aluminum–garnet (Nd:YAG) at a wavelength of 1064 nm^61^, ^62^; however, this method can have the adverse effect of potential risk of DNA damage from ultraviolet wavelength. Thereafter, erbium:YAG laser, which operates at a wavelength of 2900 nm was introduced^63^, ^64^; however, it required direct contact using laser fiber, raising concerns related to damage and contamination. Furthermore, holmium:yttrium–scandian–gallium–garnet (Ho:YSGG)^65^, ^66^ laser using at 2100 nm wavelength exhibited different absorption behavior in water than earlier lasers; moreover, it required quartz slides. Several studies have applied ArF,^67^ KrF,^68^ XeCl^69^; however, they were impracticable. Finally, a non‐contact 1480‐nm diode laser‐induced micro‐drilling procedure has been introduced^70^; six types of lasers are now available for assisted reproductive technology based on infrared‐emitting diodes currently in the market.^71^ The use of this approach is helpful for shortening the duration and reducing the complexity of zona breaching and TE biopsy. However, the potential disadvantages of this approach have not been clarified. For example, the toxicity of heat diffusion, which must remain below 100°C, has not been determined.

Does the laser‐assisted biopsy introduce mosaic or chaotic changes to biopsied cells? This discussion has just started, and there is also a controversy. At American Society for Reproductive Medicine meeting in 2017, Kelka et al^72^ concluded that laser‐assisted trophectoderm biopsy (TEB) did not have an impact on DNA profiles, however, at the European Society of Human Reproduction and Embryology meeting in 2019, Herrero et al^73^ concluded that laser assisted TEB may increase the risk of mosaicism. This mechanism may not be complicated; the number of laser shots and power of laser pulse may influence the induction of mosaicism. However, sample quality and biopsy technique may influence the results. Further investigation is needed to assess this mechanism, and it is necessary to conclude whether flicking method is superior to pulling method (Figure 1D).

#### 1.3.5 Tubing procedure

Blastocoel fluid and blastocyst culture medium contain cell‐free DNA, and PGT‐A protocols have already been established using liquid biopsy technique^74^, ^75^ and culture media.^76^, ^77^, ^78^, ^79^ The efficacy of these minimally invasive PGT approaches is controversial,^80^, ^81^, ^82^, ^83^ and regardless of diagnostic accuracy of PGT. These data showed biopsied TE samples are contaminated with higher concentrations of cell‐free DNA. Therefore, practitioners should be careful to avoid this contamination and may apply a washing step to improve purity. Furthermore, retrieval of fragmented TE cells may increase inconclusive results. Accordingly, practitioners should avoid suction of fragmented TE during the biopsy procedure. Unamplified samples mainly result from the absence of TE cells, and recent data have shown that the rate is <1.5% when analyzing 5 TE cells using a whole‐genome amplification kit. To decrease the rate of “No result” is very important because it is associated with a higher probability of error during the tubing procedure. We may avoid this error to a certain degree with a technique to confirm that a mass of TE cells is not brought out in the pipette with back current after sample loading in the PCR tube. By expelling the content of the capillary pipette into an empty dish, we can determine the remaining TE cells in the solution. If TE cells are retrieved in the dish, it is recommended to try sample loading again. Actuary we experienced 2 cases of re‐loading in 149 TE biopsies and these 2 blastocysts were completely analyzed, and we consequently achieved a 100% (149/149) of diagnostic rate.^5^

#### 1.3.6 Timing of cryopreservation after TE biopsy

There are two transfer strategies for euploidy embryos currently in clinical practice: vitrified‐warmed or fresh embryo transfer. Biopsy of blastomeres on day 3 permits analysis of diagnostic outcomes before day 5 using advanced molecular biology techniques. Several reports have described PGT‐A using the fresh transfer strategy.^47^, ^84^, ^85^ However, the use of TE biopsy and vitrified‐thawed blastocyst transfer protocols is increasing worldwide.^86^, ^87^, ^88^, ^89^, ^90^ On the other hand, the optimal timing of vitrification after TE biopsy has not been thoroughly discussed. In one study, the optimal time was reported to be 10‐15 minutes,^91^ whereas another study reported that 30 minutes or less was the optimal.^92^ In contrast, another report showed that the optimal time was >3 hours to enable blastocysts to reach re‐expansion.^93^ From our experience in a pilot study, most blastocysts are vitrified within 30 minutes after TE biopsy, enabling high rates of clinical pregnancy per transfer. However, additional clinical studies are needed to clarify the optimal protocol. In the step of euploidy blastocyst transfer after thawing, basically, we did not assume a special procedure for embryo transfer, and we performed a zona‐free transfer, similar to the routine protocol in the pilot study.^5^

## 2 CONCLUSION

The efficacy of PGT‐A is still controversial, and various factors, including diagnostic accuracy (sensitivity and specificity) and biopsy and tubing procedures, affect reproductive outcomes. In particular, Paulson showed that PGT‐A may cause 20% embryo loss.^1^ Two TE biopsy methods were used; however, the gold standard trophectoderm (TE) biopsy procedure has not established yet. Based on our experience, zona breaching on days 5‐6 after insemination has greater benefits than that on days 3‐4. Moreover, biopsy of herniated TE samples simplifies and shortens the procedure, and a non‐assisted hatching protocol may make the approach more complex if blastocysts collapse during TE suction. Recent studies have indicated that excess laser pulses may induce a higher frequency of mosaicism; thus, a rapid flick within the minimum range and fewer laser pulses are recommended. Smaller zona holes produce thinner TE cells at the hatching point, which may facilitate biopsy. Five to 10 TE cells are recommended for biopsy to decrease amplification failure. Achieving proficiency in these various techniques (Table 2) is expected to lead to optimal results. Since, biopsy techniques have not been discussed extensively in the literature, we hope that this review will facilitate further advancements in the study of this procedure.

**Table 2:** Critical points for trophectoderm biopsy

Timing of zona breaching: day3‐4 or day5‐6
Zona hole size: small or large
How many cells biopsied?
Pipette size: 20‐40 mm (commercially available)
Timing of TE sampling: herniation or direct suction
Biopsy method: pulling or flicking
Size of laser pulses, and times of the laser shot
Condition of the washing step of biopsied TE cells
Tubing procedures (sample loading)

## CONFLICT OF INTEREST

The authors declare no conflict of interest.

## HUMAN AND ANIMAL RIGHTS

This article does not contain any studies with human and animal subjects performed by the any of the authors. This review focused on the literatures previously published and it did not contain ethical issues with human subjects; thus, there was no need for the study to be evaluated by an ethical committee.
